# Treatment of Dilated Stomach

**Published:** 1893-11-25

**Authors:** 


### EDINBURGH ROYAL INFIRMARY.

### Treatment of Dilated Stomach.

It is unnecessary to mention in any detail the causes of gastric dilatation, suffice to say that it occurs in the atonic forms of dyspepsia, following repeated attacks of catarrh, and is frequent in cancer of the pylorus as well as the rarer condition of fibrous structure of that orifice from the healing of a gastric ulcer. In all of these, and most markedly in the obstructive forms, the stomach dilates, and thus adds to the difficulty of passing on its contents into the duodenum, while along with this there is usually some failure of digestive power from alterations in the composition of the gastric juice. It is obvious that under these circumstances the digestive act is materially interfered with. The food retained for an abnormally long time is imperfectly subjected to the mechanical part of the stomach's functions, while the chemical action of the gastric juice is also deranged.

In consequence, under the influence of numerous yeasts and other organisms of a low type, the contents undergo fermentation with the formation of acids of the fatty series, more particularly acetic, lactic, and butyric, and the liberation of a considerable quantity of carbonic acid gas.

The stomach thus becomes filled with a mixture of gas and highly irritating fluid, which may be felt splashing beneath the hand on palpating the abdomen, and is a source of great discomfort to the patient, which increases till relieved by vomiting or the stomach tube. In such cases it is necessary to interdiet farinaceous foods, fat, alcohol, and tobacco, the patient being fed at short intervals on small quantities of nourishing food, preference being given to a nitrogenous, but not too liquid diet. Milk in moderate quantity, eggs, fish, fowl, scaped and lightly cooked meat may be given. In bad cases it may be necessary to start with predigested food or enemata. In mild cases medicinal treatment may be confined to bismuth, rhubarb, and soda, with some carminative, or occasionally freshly-prepared charcoal. When more severe it is necessary to give some anti-fermentative substance such as carbolic acid, creasote, thymol, or permanganate of potash. Great relief may also be given by regularly washing out the stomach by means of a syphon arrangement, a filter funnel being connected with the ordinary rubber stomach tube by a yard of rubber tubing.

In passing the tube for the first time, one may give a small piece of cotton wool dipped in a dilute solution of cocaine for the patient to suck, but this is rarely necessary. The tube is lubricated with a little glycerine, or simply dipped in milk; and, holding it like a pen in the right hand, it is guided into the œsophagus with the left forefinger, and the patient directed to swallow it. A little tepid water is then introduced into the funnel, which is slightly elevated, so as to allow some of it to enter the stomach, and on depressing the funnel the syphon action is set up, and the stomach can be emptied, aided by slight pressure on the abdomen. This is repeated till the fluid remains clear, then tepid soda solution, Vichy, or Carlsbad water may be introduced. This may be done daily, or less frequently, as required, the most convenient time being about an hour before the chief meal of the day.

This method of treatment is suitable for a large nnmber of cases, including malignant stricture of the pylorus, but is contra-indicated by a history of recent hæmatemesis. It is often followed by rapid improvement, and even in cancer the patient may gain weight during its use, while perhaps no other method is so effectual in relieving the disagreeable symptoms of gastric fermentation.

In the simpler and more curable cases, where there is no obstruction of the pylorus, it is advisable also to endeavour to promote the return of the stomach to its normal size. This may be done by the application of the faradic current over the stomach while empty, and the use of nux vomica or ergot. The conditions suitable for pylorectomy and other more recent surgical procedures are not as yet, perhaps, clearly defined, but it is possible that they may become serviceable in the treatment of dilatation of the stomach depending on organic obstruction.

